# Supplementary figures and images for: Haspin kinase modulates nuclear architecture and Polycomb-dependent gene silencing
Source: PLoS Genet. 2020 Aug 4;16(8):e1008962. doi: 10.1371/journal.pgen.1008962 (PMC7428214; doi:10.1371/journal.pgen.1008962)

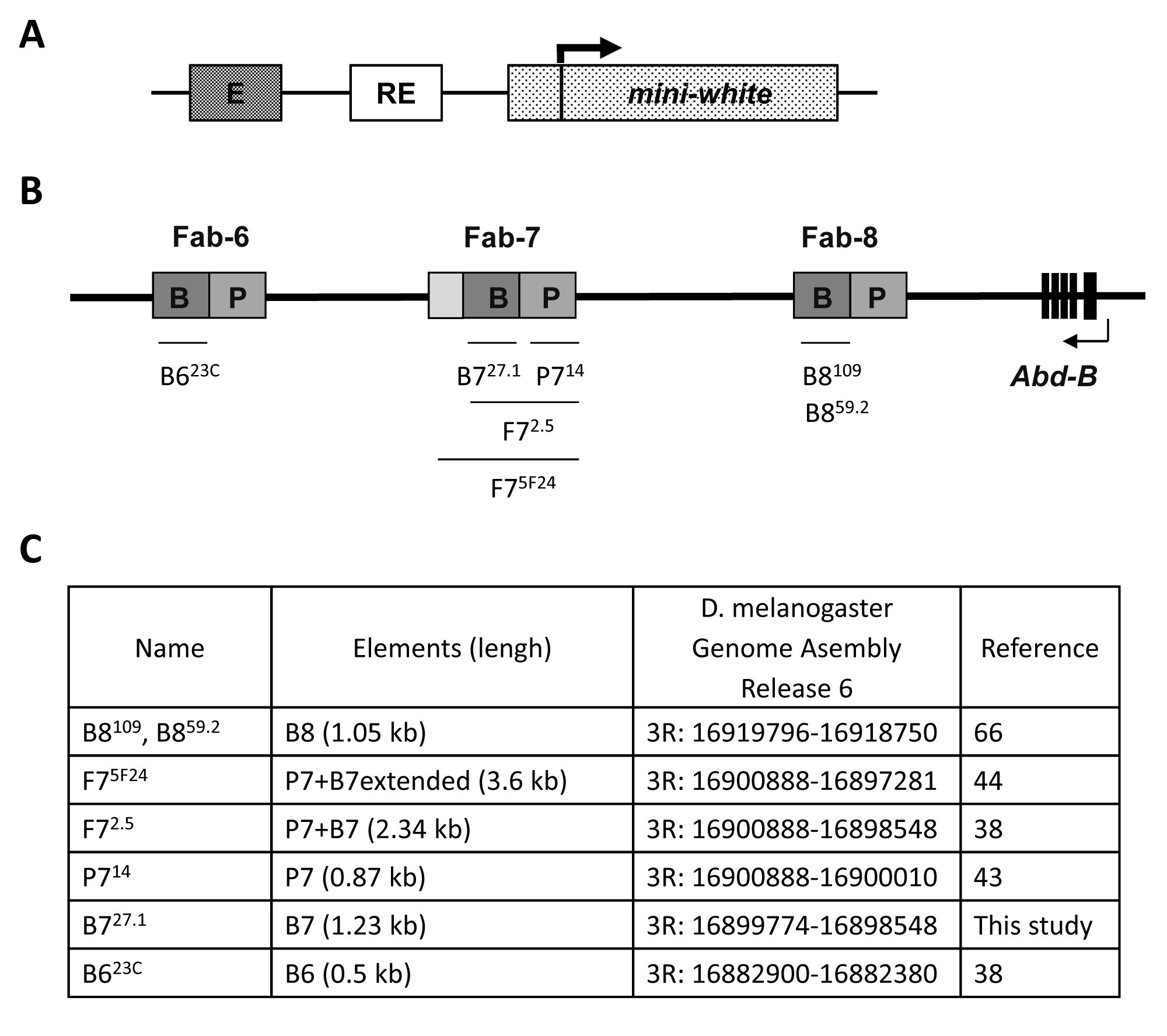

Supplement: S1 Fig — A) Diagram of the reporter used in enhancer-blocking assays. E corresponds to White enhancer sequences (X: 2798339–2796777) that contain the eye enhancer [70]. RE indicates the different regulatory elements and mini-white is the reporter gene. B) Scheme of Abd-B genomic region with the regulatory elements used in this study. B indicates boundary/insulator element and P indicates PRE. C) Locations of the fragments corresponding to the regulatory elements in the different transgenic lines are indicated. (TIF) [file pgen.1008962.s001.tif]

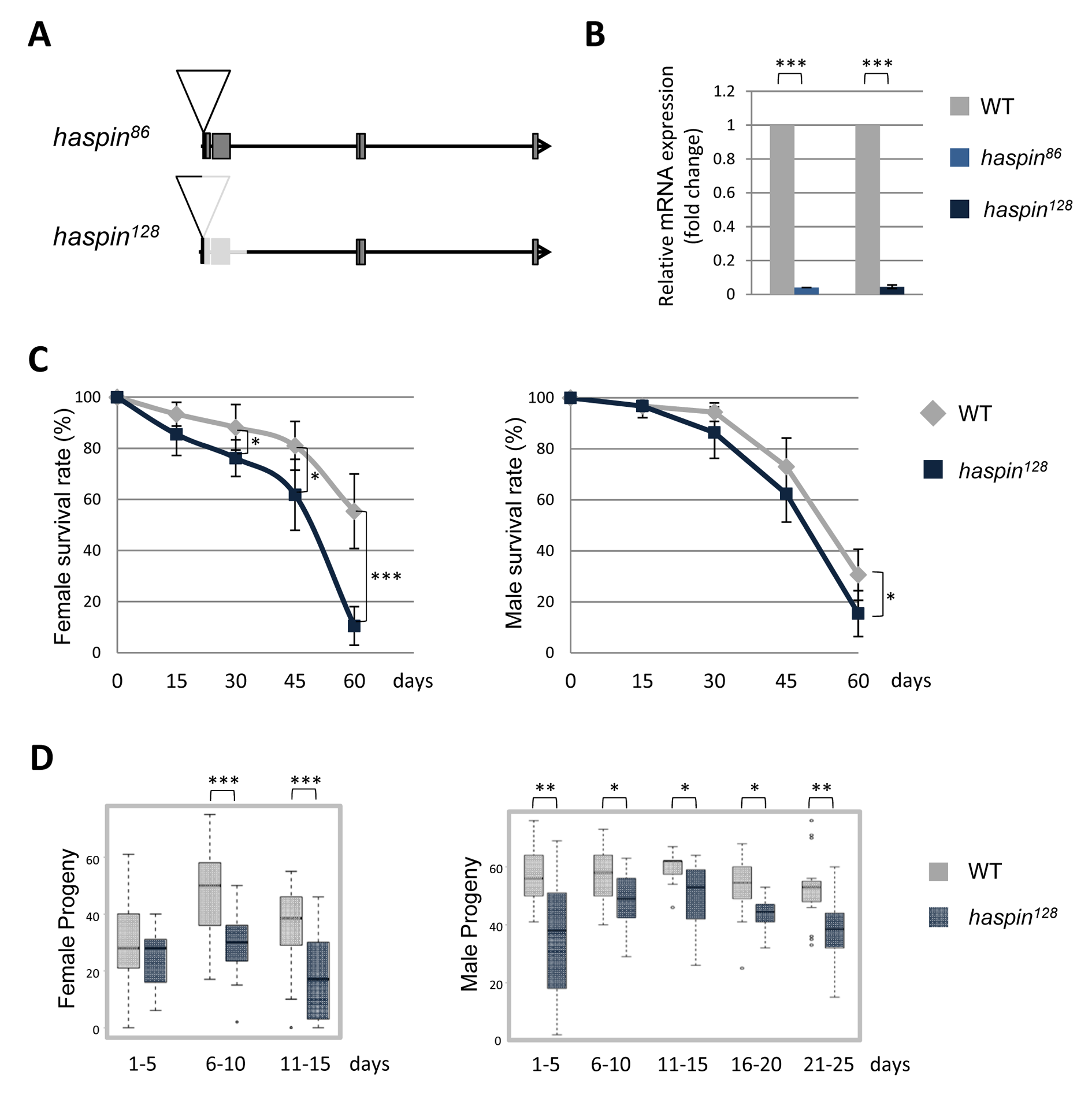

Supplement: S2 Fig — A) Scheme of haspin genomic organization in haspin86 and haspin128 mutant lines. Location of the P element is indicated by a triangle and deleted sequences in line haspin128 are indicated in grey. B) Haspin transcriptional levels normalized to Actin5C as fold changes relative to control in haspin86 and haspin128 mutant larvae. n = 3, means and s.d. are shown. C) Survival curves for wild-type and haspin128 adult flies. n≥5, means and s.d. are shown. D) Fertility test: progenies of wild-type and haspin128 flies of the indicated ages were counted and plotted (n≥20). In these tests haspin128 females did not survive more than 15–20 days. Statistical significance (*p<0.05, **p<0.01 and ***p<0.001) was determined by Student’s t-test (panels in B and C) or Wilcoxon test (panels in D). (TIF) [file pgen.1008962.s002.tif]

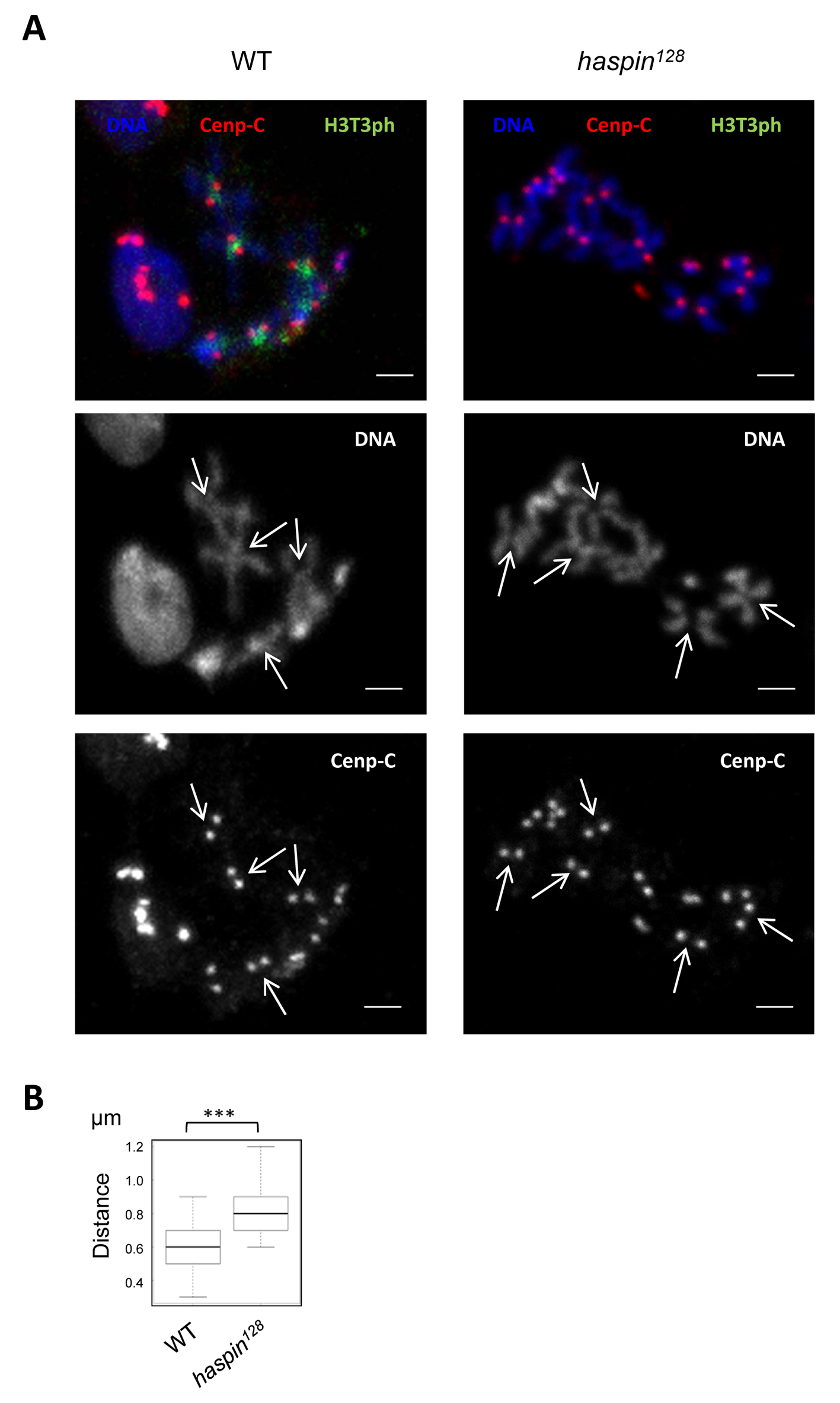

Supplement: S3 Fig — A) Immunolocalization at higher magnification of H3T3ph (green) and Cenp-C (red) on chromosome spreads prepared from wild-type and haspin128 Drosophila larval brains arrested in mitosis with colcemid. DNA is stained with DAPI (blue). Scale bars are 2 μm. B) Box plot showing quantification of inter-kinetochore distances in wild-type and haspin128 chromosome spreads. The inter-kinetochore distance was measured using the centromere marker Cenp-C in over 15 chromosomes. Distance was determined by drawing lines which length was calculated using the imageJ software. ***p<0.001 as determined by Wilcoxon test. (TIF) [file pgen.1008962.s003.tif]

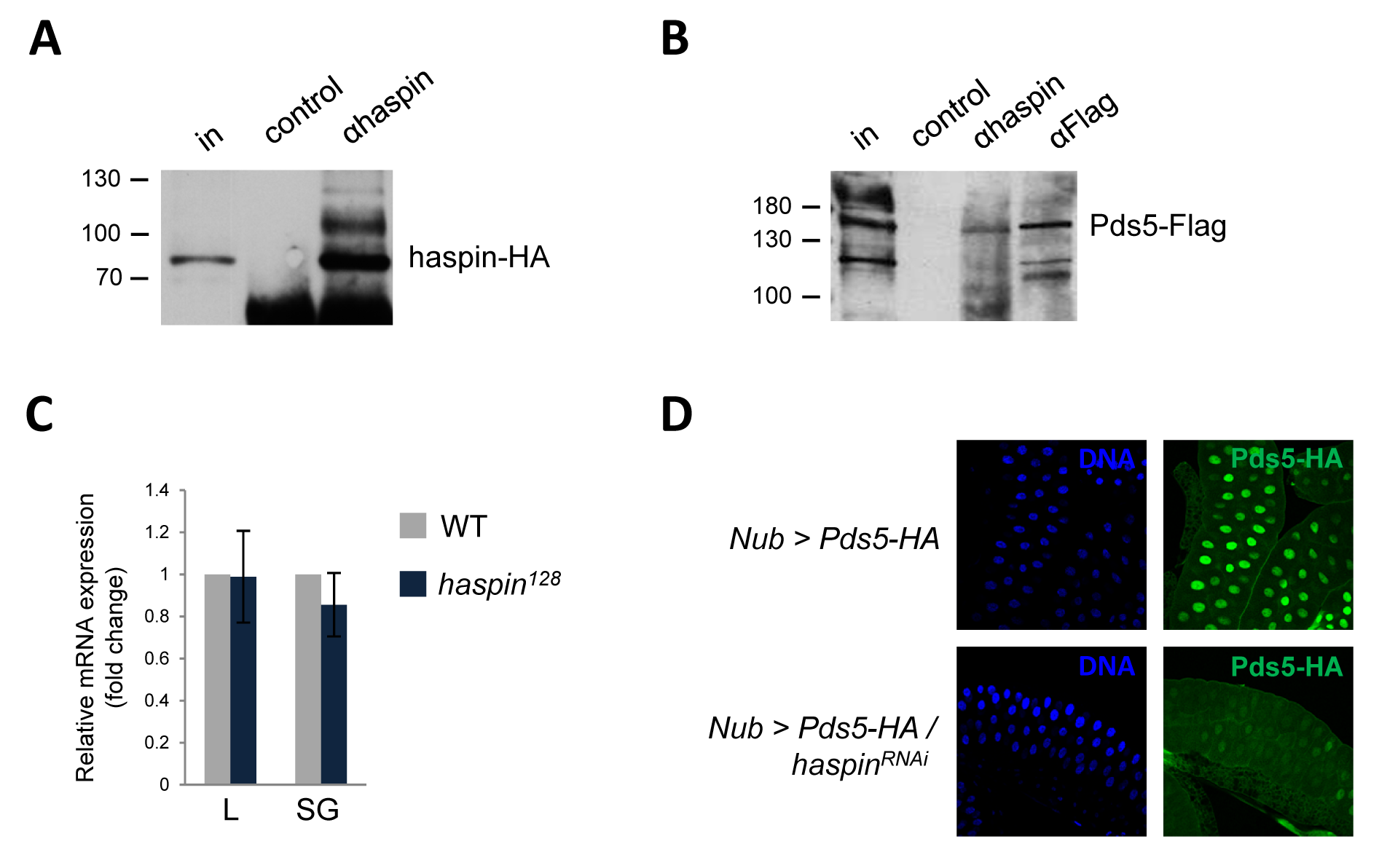

Supplement: S4 Fig — A) Western blot analysis using αHA of salivary gland extracts from larvae that express haspin-HA under the control of Actin5C promoter that were subjected to immunoprecipitation with αhaspin. Input corresponds to 10% of the immunoprecipitated material. B) Western blot analysis using αFlag of salivary gland extracts from larvae that express Pds5-HA-Flag under the control of nubbin promoter that were subjected to immunoprecipitation with αhaspin or αFlag. Input corresponds to 10% of the immunoprecipitated material. C) Pds5 transcriptional levels normalized to Actin5C as fold changes relative to control in haspin128 mutant Drosophila third-instar larvae (L) and salivary glands of third-instar larvae (SG). n = 3, means and s.d. are shown. D) Drosophila salivary glands of third-instar larvae that express Pds5-HA under the control of nubbin promoter in wild-type (upper panels) or haspin RNAi background (lower panels) immunostained with antibodies against HA. DNA is stained with DAPI. (TIF) [file pgen.1008962.s004.tif]

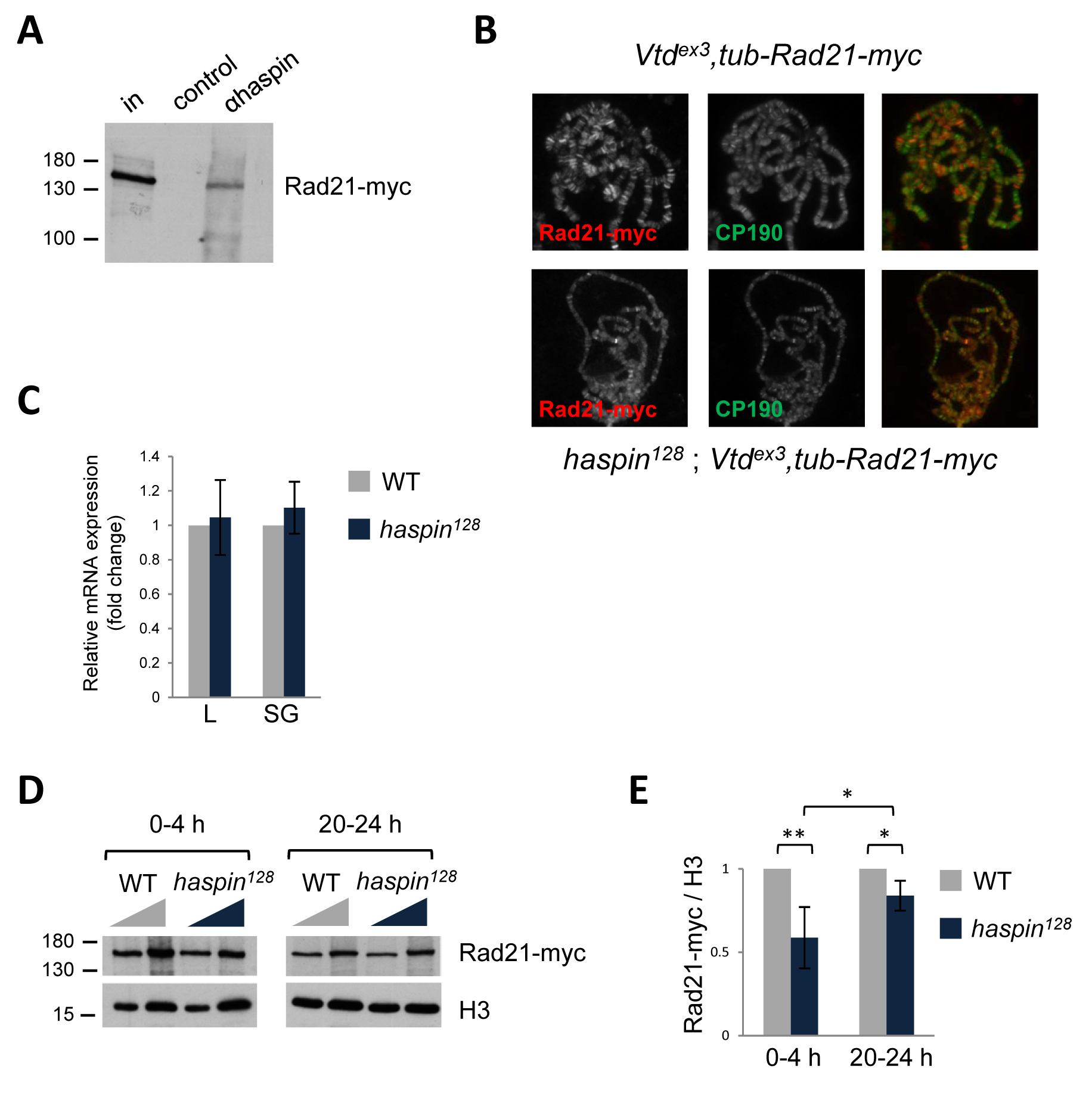

Supplement: S5 Fig — A) Western blot analysis using αmyc of salivary gland extracts from larvae that express ubiquitously Rad21-myc in a Rad21 mutant background that were subjected to immunoprecipitation with αhaspin. Input corresponds to 10% of the immunoprecipitated material. B) Representative polytene chromosome spreads from salivary glands of third-instar larvae that express Rad21-myc, under the control of tubulin promoter in a Rad21 mutant background (Vtdex3), in control (upper panels) or haspin128 mutant background (lower panels) immunostained with antibodies against myc (red) and CP190 (green). C) Rad21 transcriptional levels normalized to Actin5C as fold changes relative to control in haspin128 mutant Drosophila third-instar larvae (L) and salivary glands of third-instar larvae (SG). n = 3, means and s.d. are shown. D) Western blot analysis using αmyc (upper row) of chromatin extracts from Drosophila embryos from 0–4 h (left panel) and 20–24 h (right panel) after egg laying that express Rad21-myc in a Rad21 mutant background (Vtdex3) in control or haspin128 mutant backgrounds. Antibodies to H3 were used for the loading control (bottom row). E) Rad21-myc protein levels normalized to H3 in chromatin extracts of control or haspin128 mutant Drosophila embryos. Error bars are s.d. of three independent biological replicates. Differences in chromatin associated Rad21 are statically significant (*p<0.05 and **p<0.01 as determined by Student’s t-test). (TIF) [file pgen.1008962.s005.tif]

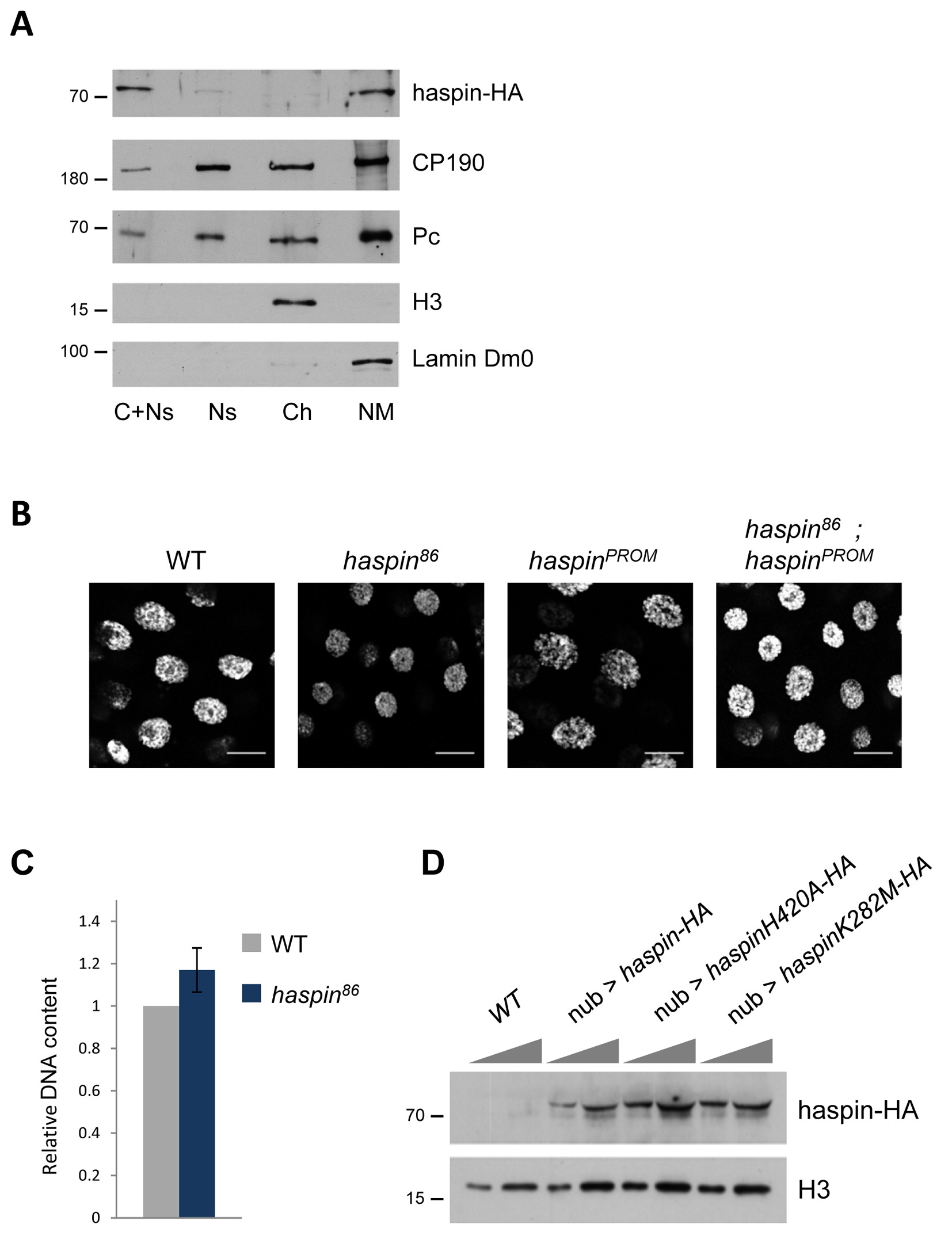

Supplement: S6 Fig — A) Biochemical fractionation of Drosophila embryos that express haspin-HA under the control of Actin5C promoter. Aliquots of cytoplasm + nuclear soluble (lane 1), nuclear soluble (lane 2), chromatin (lane 3) and nuclear matrix (lane 4) fractions were subjected to SDS-PAGE and immunoblotted with the indicated antibodies. B) Immunostaining of Drosophila salivary glands with DAPI in larvae of the indicated genotypes. Scale bars represent 50 μm. C) Relative DNA content in wild-type and haspin86 Drosophila larval salivary glands. n = 3, means and s.d. are shown. D) Western blot analysis of larval salivary gland protein extracts of control and overexpression of either wild-type protein (nub > haspin-HA) or mutated proteins in the kinase domain (nub > haspinH420AHA and nub > haspinK282MHA) using antibodies to HA (upper row). Antibodies to H3 were used for the loading control (bottom row). (TIF) [file pgen.1008962.s006.tif]

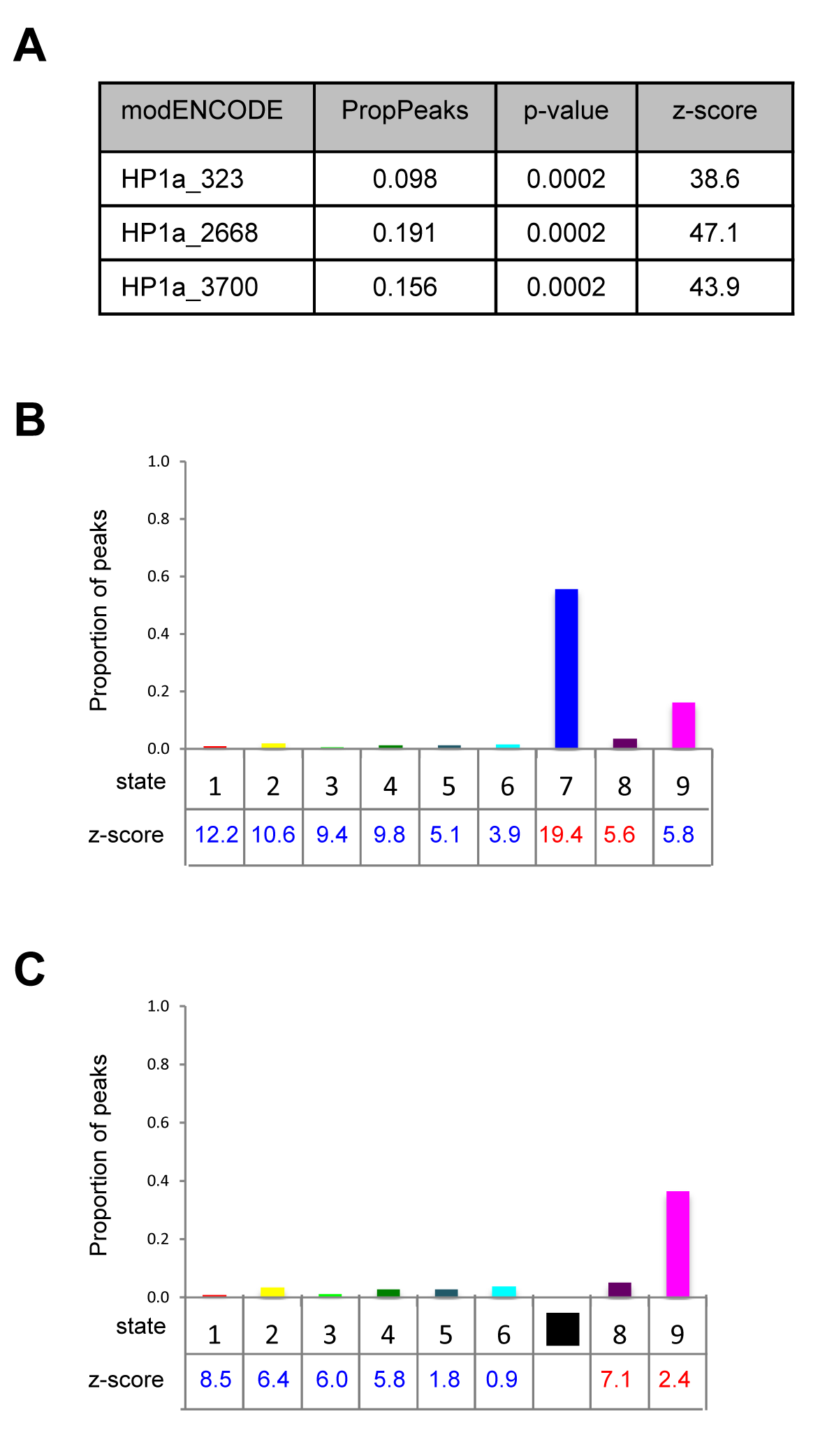

Supplement: S7 Fig — A) Proportion of H3T3ph peaks that overlap modENCODE HP1a enriched regions. Association was analyzed using overlap permutation tests with the overlapPermTest function from the regioneR package version 1.14.0 using 5000 permutations and default options. The z-score numerical measurement indicates the strength of the association. B) Proportion of H3T3ph peaks in the 9 chromatin states characterized by [34]. State 1 (red) active promoters and transcription start sites; state 2 (yellow) transcript elongation; states 3 and 4 (light and bright green) regulatory regions; state 5 (green-blue) active male X chromosome; state 6 (light blue) PcG regions; state 7 (dark blue) centromeric heterochromatin and chromosome 4; state 8 (purple) other heterochromatin; state 9 (pink) other silent domains. z-score in permutation tests is indicated below (blue and red indicate negative and positive values respectively). C) Proportion of euchromatic H3T3ph peaks, which were defined as those not overlapping with chromatin state 7, in chromatin states characterized by [34]. (TIF) [file pgen.1008962.s007.tif]

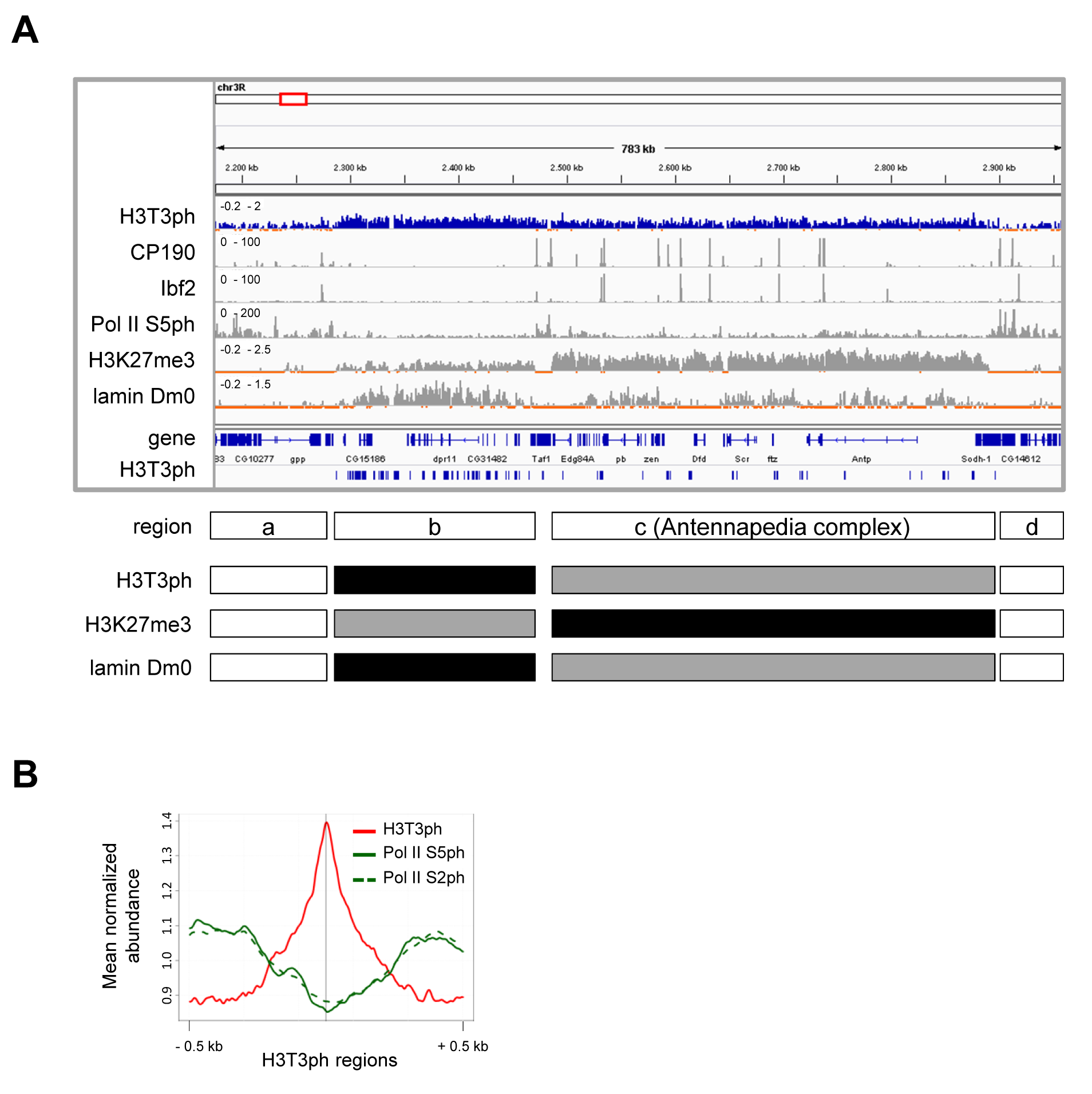

Supplement: S8 Fig — A) ChIP-seq data for H3T3ph over a region of 800 kb in chromosome 3 that contains the Antennapedia complex. Binding profiles of CP190 and Ibf2 [66], Pol II S5ph and H3K27me3 (modENCODE data) and lamin Dm0 [69] are depicted. High, low and no signal for H3T3ph, H3K27me3 and lamin Dm0 are indicated below by black, grey and white bars respectively. B) Whole genome colocalization of H3T3ph with Pol II S2ph/S5ph. (TIF) [file pgen.1008962.s008.tif]

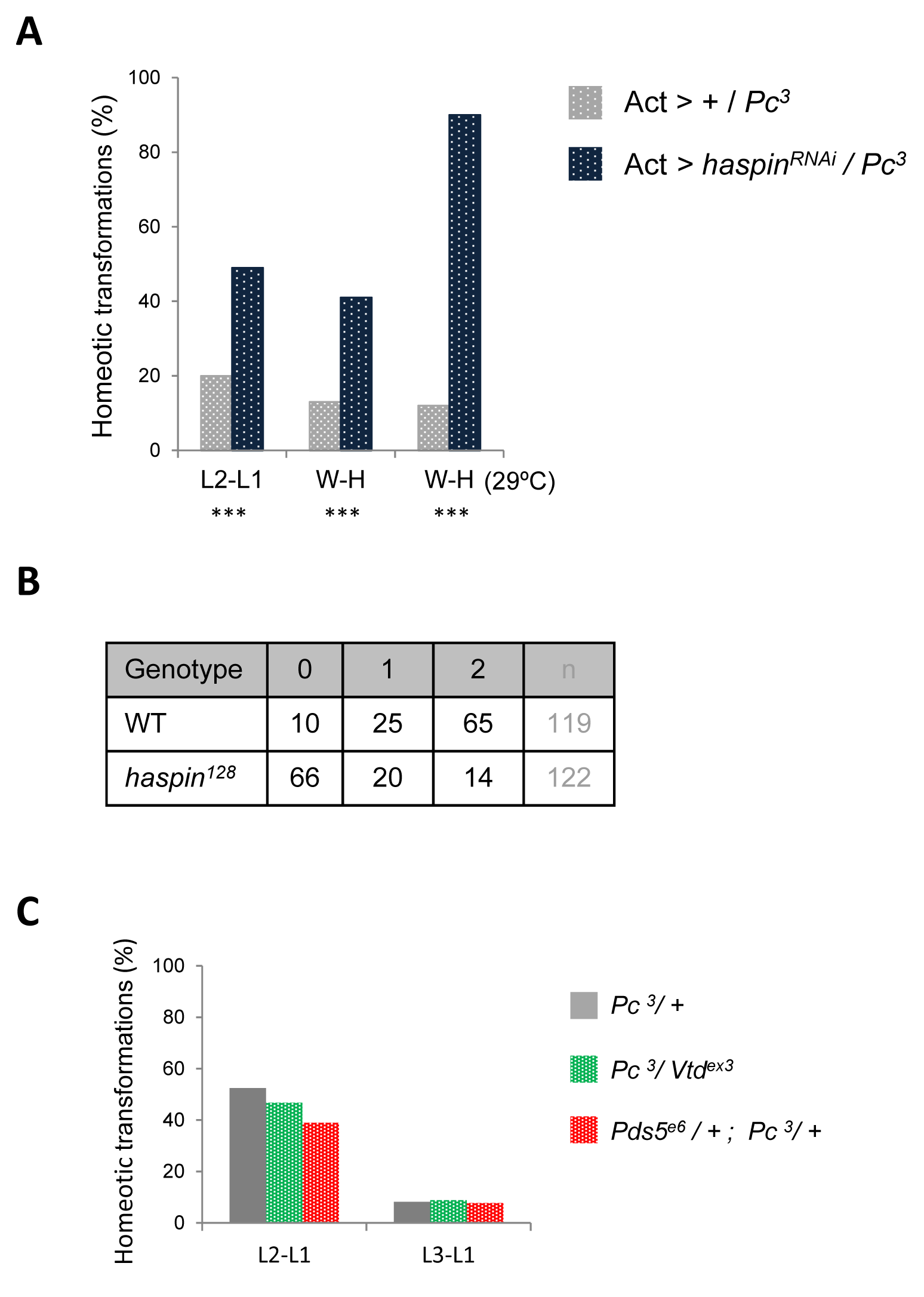

Supplement: S9 Fig — A) Frequencies of male L2-L1 and female W-H homeotic transformations in a Pc3 heterozygous mutant background either expressing or not haspinRNAi under the control of the Actin5C promoter at 25°C. Increasing temperature to 29°C caused male lethality and only female W-H transformations were scored. n = 3, over 50 and 8 individuals scored at 25°C and 29°C respectively. ***p<0.001 as determined by z-test. B) The percentage of homozygous F75F24 transgenic females at 25°C showing normal wings (0) and wing blade destruction in one (1) or both (2) wings in wild-type and haspin mutant flies is presented. n indicates number of females scored. C) Frequencies of homeotic transformations in flies heterozygous for Pc3 mutation in either wild-type or heterozygous mutant backgrounds for cohesin complex components. n = 3, over 50 individuals scored. No significant differences as determined by z-test. (TIF) [file pgen.1008962.s009.tif]
