# Supplementary material for: Haspin kinase modulates nuclear architecture and Polycomb-dependent gene silencing
Source: PLoS Genet. 2020 Aug 4;16(8):e1008962. doi: 10.1371/journal.pgen.1008962 (PMC7428214; doi:10.1371/journal.pgen.1008962)
Supplement: S1 Table — (DOCX) [file pgen.1008962.s010.docx]

**Table S1.** Primers used in RT-qPCR and ChIP-qPCR

| Haspin F  RT-qPCR | CTGGTCATCGTTTTCACCCA |
| --- | --- |
| Haspin R | CCTGTGAACTTTCGTATTGATGC |
| Actin5C F | GCGCGGTTACTCTTTCACCA |
| Actin5C R | ATGTCACGGACGATTTCACG |
| Abdominal-B F | TTACGGCTCCGGGTACTACG |
| Abdominal-B R | GGGTAAGGATAGGCGAACATGT |
| Pds5 F | GCCGTTGCGAATTTCCAAAA |
| Pds5 R | CATGATTGACTGTTCCGCCG |
| Rad21 F | TCAAGGAGACTCAACGCCAG |
| Rad21 R | GTCGTGATCCATCTCGCCAA |
| Pointed F  ChIP-qPCR | TCCATGTGTATGCGAGCGAG |
| Pointed R | CGAGTGCGGGACTTGTGTAT |
| Mcp C | TACACATAATACATGGCCGCG |
| Mcp T | AGGCAGCCATCAATGTTGC |
| Fab-7 C | GGAATACCGCACTGTCGTAGG |
| Fab-7 T | GCAGCCATCATGGATGTGAA |
| Fab-8 C | GCTTCCTACGGCATTTTTCT |
| Fab-8 T | GAATGGAACTCTTCGCTTGC |
| A prom F | GCATTAATTAGTCACTGCGCTTG |
| A prom R | GTTGGTGCGGCTCTCTTGT |
| B prom F | GGGCCATAAAACCCAAAATAA |
| B prom R | GACAAAGCCGCAGCGAAT |
