# Supplementary material for: Haspin kinase modulates nuclear architecture and Polycomb-dependent gene silencing
Source: PLoS Genet. 2020 Aug 4;16(8):e1008962. doi: 10.1371/journal.pgen.1008962 (PMC7428214; doi:10.1371/journal.pgen.1008962)
Supplement: S1 Text — (DOCX) [file pgen.1008962.s011.docx]

**Bioinformatics analyses of ChIP-Seq data**

For H3T3ph data, Illumina 50bp Single End FastQ data of Immunoprecipitated and Input DNA were aligned against the *dm3* genome using Bowtie 0.12.5 [1], using options *–v2 –m1*. Downstream analyses of aligned reads were performed using R 2.15 and Bioconductor [2]. The *htSeqTools* v.1.4.0 package [3] was used to detect and clean aligned data from overamplification artifacts using the *filterDuplReads* function with a False Discovery Rate of 0.01. Strand shift bias was corrected using the *alignPeaks* function with default settings. Enriched H3T3ph sites were detected with the *enrichedRegions* function using the options *minReads=10* and selecting as enriched regions those with a *Benjamini-Hochberg* pvalue < 0.05, and were annotated to overlapping and closest *Drosophila melanogaster* dm3 genomic features (Ensembl April 2014) using the *AnnotatePeakInBatch* function from the *ChIPpeakAnno* package [4] with the options *output='both', select='all', maxgap=0, PeakLocForDistance='middle', FeatureLocForDistance='TSS'*. The *rtracklayer* package 1.18.2 [5] was used to generate tracks for normalized IP/Input H3T3ph signal across the genome.

Additional quality control of raw and aligned data were performed using FastQC version 0.10 (<http://www.bioinformatics.babraham.ac.uk/projects/fastqc/>).

In order to assess whole genome colocalization of H3T3ph with CP190/Ibf1/Ibf2 (NCBI GEO samples GSM1133263, GSM1133264 and GSM1133265), HP1a (modENCODE), Lamin Dm0 (NCBI GEO sample GSM509086), Pol II S2ph/S5ph (NCBI GEO samples GSM593411 and GSM593412), and H3K27me3 ChIP-Seq data (NCBI GEO sample GSM480157) original data was downloaded and normalized signal was computed using custom R scripts and compared using the *regionsCoverage* and *plotMeanCoverage* functions from the *htSeqTools* package. Whole genome overlap of H3T3ph enriched regions with those defined by the 9-state chromatin model in S2 cells [6] was computed in R. Statistical assessment of the overlap between H3T3ph (Whole genome and Euchromatin specific regions) and the 9 states of chromatin was performed using the overlapPermTest function from the regioneR package version 1.14.0 using 5000 permutations and default options. H3T3ph Euchromatin specific regions were defined as those not overlapping with chromatin state 7. The same steps were performed in order to assess overlap between H3T3ph and modENCODE S2 HP1a binding sites.

**References**

1. Langmead B, Trapnell C, Pop M, Salzberg SL (2009) Ultrafast and memory-efficient alignment of short DNA sequences to the human genome. Genome Biol 10: R25.

2. Gentleman RC, Carey VJ, Bates DM, Bolstad B, Dettling M, et al. (2004) Bioconductor: open software development for computational biology and bioinformatics. Genome Biol 5: R80.

3. Planet E, Attolini CS, Reina O, Flores O, Rossell D (2012) htSeqTools: high-throughput sequencing quality control, processing and visualization in R. Bioinformatics 28: 589-590.

4. Zhu LJ, Gazin C, Lawson ND, Pages H, Lin SM, et al. (2010) ChIPpeakAnno: a Bioconductor package to annotate ChIP-seq and ChIP-chip data. BMC Bioinformatics 11: 237.

5. Lawrence M, Gentleman R, Carey V (2009) rtracklayer: an R package for interfacing with genome browsers. Bioinformatics 25: 1841-1842.

6. Kharchenko PV, Alekseyenko AA, Schwartz YB, Minoda A, Riddle NC, et al. (2011) Comprehensive analysis of the chromatin landscape in Drosophila melanogaster. Nature 471: 480-485.
