# Supplementary material for: Haspin kinase modulates nuclear architecture and Polycomb-dependent gene silencing
Source: PLoS Genet. 2020 Aug 4;16(8):e1008962. doi: 10.1371/journal.pgen.1008962 (PMC7428214; doi:10.1371/journal.pgen.1008962)
Supplement: Attachment — Submitted filename: Response to reviewers.docx [file pgen.1008962.s013.docx]

Dear referees,

I apologize for the long time we spent to perform the experiments and send a revised manuscript but it has been a hard time for us. In December 2019, I went through major surgery and the two students that did part of the work finished her PhD and left the lab. When I started to recover, strong restrictions to access the lab due to the COVID-19 pandemic have been implemented and we have experienced challenges to address some of your concerns. Please find below a detailed answer to your questions and thank you for your consideration.

Referee 1:

Sometimes, Suppression of enhancer blocking activity of insulators can be sensitive to the background. The authors describe a rescue construct for Haspin (Haspin-prom). If they have the rescue construct recombined on a chromosome carrying Haspin (86) it should be a simple cross to document reversion of the Suppressor effect. I usually hate to ask for additional experiments and the authors showed that knocking down Haspin by RNAi also leads to the suppression of Fab-7-mediated enhancer blocking activity. So my suggestion is not mandatory, but if the rescue experiment could be added, it would be perfect.
We agree with the referee and we have tried to analyze reversion of the eye color phenotype with the rescue construct. To do that the white marker of the attB plasmid used to obtain the rescue construct haspin^PROM^ was eliminated via Cre recombinase-mediated excision and we set up crosses to analyze Fab7 enhancer-blocking in haspin^128^ mutation with haspin^PROM^ rescue construct. However, we have not been able to analyze rescue since almost no flies bearing the two constructs in the mutant background were obtained, even though a high number of individuals were scored.

At page 5 the authors should inform right away the readers that flies apparently knocked down for haspin are viable. Are the homozygotes fertile? What is known about maternal contribution? Looking at Flybase (which by the way mentions the existence of 5 uncharacterized alleles), there is a moderate presence of the mRNA in 0-2 hours embryos. The authors mention semi fertility of the female. Do these eggs develop through adulthood?
We agree with the referee that this was not clearly explained in the previous version of the manuscript. We have revised this section and the viability of haspin homozygous mutant alleles is now clearly indicated. As shown in Figure S2D, homozygous haspin mutant flies produced fewer progeny than controls indicating that fertility of both sexes is affected, although a significant number of eggs develop through adulthood and stocks can be maintained in homozygosis. We have not analyzed maternal contribution and nothing is known about that since our work is the first study of haspin in Drosophila during development.

I find difficult to monitor the increase in the distance between centromeres of mitotic chromosomes in metaphase in the Haspin mutant background (Fig.2). Somehow the green H3T3ph signal in between the cenp-C spots blur the aspect. It would be perhaps useful to display a red channel only panel for the WT chromosome.

Following referee’s suggestion in this revised version of the manuscript blue (DNA) and red (Cenp-C) only panels are shown in Figure S3. Differences in connection between chromatids can be now clearly seen in DNA panels and these differences are confirmed by measuring the distance between Cenp-C spots.

In the middle of page 7, the authors describe the generation of an antibody against Haspin protein. Have they tried staining of salivary gland? It would be interesting to correlate it with the staining detecting phosphorytaled H3T3. By the way did the authors checked if the BX-C is decorated by H3T3ph? I was not able to recognize 3R on the spread displayed in FigS4, but the quality of their squash is fine and a direct observation under the microscope would perhaps allow to find the BX-C. I also wonder if it is possible to correlate the H3T3ph chipseq data with the staining on salivary gland.

We tried haspin antibodies in immunostaining experiments in salivary glands among other Drosophila tissues but they did not give any specific signal, likely due to technical issues.

We are not specialist in polytene chromosomes and, although differences in signal between control and haspin mutant background are very clear, the quality of the H3T3ph signal in wild-type chromosomes did not allow us to correlate staining on polytene chromosomes with the H3T3ph ChIP-seq data. Besides, our ChIP-seq data show that the BX-C is an H3T3ph low enriched region and, therefore, should not correspond to a clear band in polytene chromosome spreads.

Bottom of page 7 the authors mention no apparent change of Rad21 binding to salivary gland giant chromosome in the absence of Haspin. But it seems to me that there is a decrease in intensity and in the number of sites decorated by Rad21 in haspin mutant background. In general, I also find that the quality of the squashes generated in Haspin mutant background is lower, with thinner and less-well spread chromosomes. Is that a general trend or a coincidence? The authors should check if Rad21 expression is not decreased in haspin mutant background.

We agree with the referee. The quality of the squashes generated in haspin mutant background is in general lower, with thinner and less-well spread chromosomes, likely reflecting the role of haspin in modulation of chromatin organization during interphase. However, it is difficult to unambiguously demonstrate this phenotype due to the high degree of technical variability in polytene chromosome squashes. Therefore, we decided not to discuss this point.

With respect to Rad21 expression we have analyzed Rad21 mRNA levels in haspin mutant background and we did not found significant changes with respect to control. This is shown in Figure S5C in the revised version of the manuscript.

I am not entirely sure of what to do with the observation that Abd-B expression increases in the haspin(86) mutant background. If this increase originates from a weakening of the Pc-G mediated repression, one should also observe ectopic activation leading to slight gain-of-function phenotype such as the appearance of pigmentation on the 4th male tergite. I imagine that the authors would have noticed it. So the lack of extra-pigmentation is puzzling to me. Abd-B is hapo-insufficient (this is why it is written with an uppercase A). Thus Abd-B/+ males have often a few bristles on the 6th sternite as well as slightly extended genitalia. I wonder if this haplo-insufficiency can be compensated by introducing the haspin mutation? This could be also tested for the haplo-insufficient phenotype of Ubx mutations (Ubx/+ have slightly increase halters).

We have not observed the appearance of pigmentation on the 4th male tergite in haspin mutant background suggesting that the small increase in Abd-B expression is not enough to lead to gain-of-function phenotypes. Following referee's suggestion, we have analyzed haspin128/+;Abd-BM1/+ males but we did not observe any rescue of the A6 to A5 transformation. We have also tried to analyze rescue in haspin homozygous background but the intermediate cross we designed with males cyo/+;Abd-BM1/MKRS did not progress. We are at present producing an intermediate line with different balancers, which should allow analyzing this. However, we will not have these results before, at least, two more months, depending how the situation evolves and whether additional restrictions of work at the Institute are implemented. For this reason, and taken into account the delay we already took in answering referee's comments, we have decided to send the revised version of the manuscript without including this. We think that the impact of this additional data on the conclusions of the work would be minor. A positive result would indicate that haspin mutation by his own compensates decreased levels of Abd-B, likely due to weakened PcG-mediated repression, and the data presented already shows that haspin mutation enhances polycomb-dependent homeotic transformations indicating cooperation between haspin and polycomb in homeotic regulation.

Regarding the remark in the middle of page 13 about the difference in extra sex comb formation during the 1st three generations, it is not necessary to invoke trans-generational inheritance. It is indeed a well-known fact that Pc-G stocks accumulate modifier mutation that suppress their haplol-insufficient phenotype. By simply out-crossing, these modifiers loci are diluted out. The authors should have observed the same phenomenon occurring even with the WT chromosome 2 control.

We are thankful to the referee for his advice and we have eliminated the remark.

Finally, as a dosophilist, I am sensitive to the naming of mutations that most of the time relate to the process involved. As a matter of facts many geneticists were/are very creative in naming their favorite mutations. I am therefore curious to know why this kinase is named “Haspin”. I understand that in this case there will be no relationship between the name of the mutation and the phenotype. Given the fact that the homozygotes do not show an apparent phenotype, I suggest writing the genotype with a lowercase 1st letter “haspin”.

Name of haspin (haploid germ cell-specific nuclear protein kinase) is related to the process where this protein was first characterized, differentiation of haploid germ cells. Until now there are only two reports on Drosophila haspin (Xie et al. 2015. Cell 163:920-933 and Dattoli et al. 2020. JCB 219: e201910084), both dealing with germ stem cell differentiation. In both articles mutation of haspin was carried out with RNAi lines and neither homozygous nor heterozygous mutant lines were analyzed. Our work is the first study that characterizes haspin null mutant background and, although we have not analyzed germ stem cells, heterozygotes do not show an apparent phenotype. Thus, we have followed referee's suggestion to write the genotype with a lowercase 1st letter.

Referee 2

Major points:

1. For readers who are not fly specialists, it would be useful to describe the eye-colour change assay more clearly. In addition, it is unclear how strong the observed phenotype is compared to other regulators of this system. Can controls without the insulator element or with a knockdown of a protein with known insulator activity be carried out?

Following referee's suggestion we have described the enhancer-blocking assay in more detail in the results section. In general, transgenic lines containing constructs without an insulator element between the white enhancer and the mini-white reporter gene have red eyes since the enhancer strongly activates expression of the white gene. On the other hand, knockdown of insulator proteins results in intermediate eye-color. In this revised version of the manuscript we have introduced in Figure 1C results with the insulator protein CP190 to allow phenotype comparisons.

2. The finding of H3T3ph during interphase is novel and exciting. However, all the data to support this are in polytene chromosomes while other aspects of this study are in other cell types. Can H3T3ph be detected in interphase in other cells types or is this a polytene chromosome specific phenomenon (nuclear H3T3ph staining should be simple to observe)? In addition, is H3T3ph on polytene chromosomes enriched at the centromeres as seen in mitosis?

We have not been able to detect specific H3T3ph staining in other cell types (we observe some nuclear staining which is difficult to unambiguously assign to H3T3ph specific signal). Nevertheless, we think that this can be due to technical issues such as diffuse distribution of the mark and quality of the antibody, since visualization of protein immunostaining is easier in polytene chromosomes due to the extensive amplification of both DNA and chromosomal proteins. Besides, our ChIP-seq in S2 cells show that H3T3ph localizes not only to centromeric regions (which could be due to mitosis activated signal) but also to other heterochromatic and silenced regions in the arms of the chromosomes (see also below, minor point 9). These results are analyzed in detail in this revised version of the manuscript in the Results section, in Haspin is a suppressor of position-effect variegation subsection.

Centromeric sequences in polytene chromosomes, which are associated in the chromocenter, have been shown to be under-replicated. This can explain why H3T3ph staining at the chromocenter in our immunofluorescence assays is less intense than signals that are observed at discrete loci on extended polytene chromosome arms.

3. The evidence that Haspin really functions in interphase (other than H3T3ph on polytene chromosomes is weak). The experiment in Figure S4E is not compelling because it is very indirect and in fact shows that the extent of Rad21 localisation actually correlates with the %mitotic cells in two populations.

This experiment, now presented in Figure S5E, shows that haspin is required for cohesin binding to chromatin in Drosophila embryos indicating that haspin modulates the dynamic association of cohesin with chromatin during the cell cycle. Our conclusions on the role of haspin in interphase are not based on this experiment but on experiments showing that haspin modulates key aspects of chromatin organization during interphase: insulator activity, nuclear morphology, heterochromatin organization and polycomb-dependent gene silencing. Moreover, besides H3T3ph presence on polytene chromosomes, ChIP-seq assays show that H3T3ph localizes not only to centromeric regions (which could be due to mitosis activated signal) but also to other heterochromatic and silenced regions in the arms of the chromosomes. In the revised version of the manuscript we have also introduced new experiments that show that haspin interacts with Pds5 in interphase (Figure S4B) and it is required for the stability of the protein (Figure S4D). Finally, our results show that haspin mediates Pds5 binding to chromatin and associates with the cohesin complex in interphase.

4. The IF results in Figure 2 should be quantified to show the effects are consistent across many cells, and to give some idea of the extent of protein loss from chromosomes. Does PDS5 also disappear from chromosomes in Haspin mutant cells?

Following referee's suggestion we have performed quantifications of Pds5 IF assays that are shown in Figure S4C in the revised version of the manuscript.

We have not analyzed Pds5 in Haspin mutant cells in this work since we obtained partial levels of haspin depletion using RNAi techniques in S2 cells. We are aware that extensive investigations will be needed to decipher the relationship between haspin and pds5-cohesin complex during the cell cycle (see also below, point 5) and we will undertake them in the framework of our future work.

5. PDS5 proteins have been shown to bind to SCC1 directly. Figure 2C shows that, in the absence of haspin, PDS5 cannot bind to chromatin yet the absence of haspin does not prevent the binding of the majority of the core cohesin complex. This is interesting, but contrary to the popular model that PDS5 localises to chromatin through cohesin and Haspin is localised through PDS5. This raises the question of whether the loss of PDS5 is due to Haspin acting as a chromosome receptor for PDS5, or whether it is an indirect effect resulting from Haspin’s function in regulating cohesion. Can this be addressed For example, does haspin co-IP with PDS5? (It might also be interesting, but not essential, to know if the chromatin fraction in a haspin knockdown no longer contains PDS5 and it is instead found in the soluble fraction).

Following referee's suggestion we have performed coimmunoprecipitation assays, which are shown in Figure S4B in the revised version of the manuscript. These assays showed interaction between haspin and Pds5 during interphase. Our new data also show that total Pds5 protein levels are reduced in haspin mutant background (Figure S4D). Altogether, these results strongly suggest destabilization of Pds5 protein due to its inability to associate with haspin and chromatin and point to the interdependence between haspin and Pds5 for chromatin binding.

6. Biochemical fractionation (figure S5A) shows no Haspin in the chromatin fraction, which is somewhat surprising and perhaps should be explained/commented on.

We agree with the referee that this deserves more extensive discussion. Our biochemical fractionation assays show that haspin is found mostly associated with the nuclear matrix, although there is also some low amount of haspin in the chromatin fraction, which is not apparent in the western presented in Figure S5A since it is low exposed. While proteins that are associated with transcriptionally active chromatin are easily solubilized after nuclease treatment, haspin, insulator and polycomb proteins are tightly bound to the nuclear matrix where they colocalize with laminas (our results and previously reported studies [30,31]), suggesting that nuclear organization of these proteins and the chromatin regions they bound might contribute to their functionality. This is now commented in the Discussion section of the manuscript.

7. It would be useful to know the extent to which Haspin-HA is overexpressed. It is surprising that Haspin-HA overexpression returns nuclear size to normal in the Haspin mutant (Fig 3C) when a single additional copy of the Haspin gene is sufficient to increase nuclear size (Fig 3B).

In Figure 3B, haspinPROM construct is in homozygosis, that is two extra copies. This was not properly described in the previous version of the manuscript. Haspin polyclonal antibodies that were generated in this work did not show any specific signal in western-blots and we have not been able to analyze the extent to which haspin-HA is overexpressed. However, haspin-HA overexpression using 69B driver in haspin null mutant background slightly increases nuclear size suggesting that expression is a little higher than endogenous haspin expression.

8. The antibody used in the H3T3ph ChIP-seq experiments has not been sufficiently validated for use in ChIP-seq. It is common that adjacent modifications can prevent histone antibody binding. In this case, H3K4me3 is of particular concern and the authors make several conclusions regarding K4me3, TFIID and RNA pol II. Without validation of the antibody’s ability to bind to H3T3ph adjacent to K4me3, these claims are not supported by the presented data. We suggest that the authors carry out an ELISA or similar assay to address this issue (in fact our own data indicate that JY325 is strongly affected by H3K4 methylation).

We agree with the referee that adjacent histone modifications can prevent antibody binding in ChIP experiments and future investigations will be needed to further decipher whether or not H3T3ph never colocalizes with H3K4me and active transcription. For this reason, in the revised version of the manuscript we have moved this Figure to Supporting Information and we have further discussed this point.

9. Visual inspection of ChIP-seq data is insufficient to draw conclusions about the colocalization of H3T3ph and other factors. Bioinformatics analysis showing the statistical significance of enrichment of multiple factors in various chromatin states or extent of peak overlap should be shown to validate this.

In the revised version of the manuscript we performed bioinformatics analyses using overlap permutation tests that showed a high degree of statistically significant association of H3T3ph and HP1a binding sites. These analyses are shown in Figure S7A. We also used overlap permutation tests to statistically assess the location of H3T3ph peaks in relation to the 9 chromatin states previously characterized by Kharchenko et al. [33] as enriched in different chromatin factors and histone modifications. These analyses are now shown in Figures S7B and S7C.

10. The authors typically equate Haspin action with H3T3 phosphorylation, but the difference should be made clear. Haspin has non-kinase dependent roles, and it also should be acknowledged that Haspin is likely to have substrates other than H3T3ph (eg Wapl).

We completely agree with the referee that haspin action cannot be equated to H3T3 phosphorylation and this was not our intention. We have revised the text in order to avoid this interpretation. Our work strongly suggest that haspin participates in the functionality of silent chromatin by modulating H3T3ph and Pds5-cohesin-chromatin dynamics, though future work will be needed to characterize in detail haspin substrates and mechanisms involved.

11. Throughout, the number of repeats (n=?) should be provided in figure legends.

We have now included this information.

Minor Points:

1. There are several grammatical errors and typos. Examples include use of the word ‘being’ mid-sentence (abstract and several other sentences); commas after the word “both” are in all cases misplaced and confusing; hapin instead of haspin (p. 9), different not diferent (p.5).

Done.

2. Figure 1A legend does not match labels on the figure (B7 or F7 transgenic line)

It should be B7; this has been corrected.

3. Generally, the figure legends should provide a better description of the abbreviations used in the figures. Particular examples include figure 1 C and D

Done.

4. Molecular weight markers should be shown on the western.

Done.

5. In figure 3, details on how nuclear size is defined and quantified should be expanded. The same applies to figure S5C for relative DNA content.

We have now expanded this information in Materials and Methods section, in Quantification of immnufluorescence images and DNA content analysis subsections.

6. The use of chromatin compaction (page 8) is misleading when referring to a change in nucleus size as the data does not address whether the chromatin itself changes..

As pointed out by referee 1 the quality of the polytene chromosome squashes generated in haspin mutant background is in general lower, with thinner and less-well spread chromosomes, likely reflecting a role of haspin in chromatin compaction. However, it is difficult to unambiguously demonstrate this phenotype due to the high degree of technical variability in polytene chromosome squashes and we agree with the referee that the use of chromatin compaction is misleading when referring only to a change in nuclear size. In this revised version of the manuscript we have changed chromatin compaction by nuclear compaction.

7. In figure S6A, H3T3ph is not found in state 8 defined as “other heterochromatin” but is largely found in state 7 which is defined as “centromeric heterochromatin”. The authors should discuss these states in more detail and provide a suggestion as to why they believe this is not just the known mitotic centromeric H3T3ph signal. In addition, the authors claim a colocalization with HP1a or lamin – are these also enriched in these states?

The model generated by Kharchenko et al [33] distinguishes two states, 7 and 8, which are enriched in chromatin marks typically associated with heterochromatic regions (H3K9me2/me3 and HP1a). While state 7 corresponds to centromeric heterochromatin and chromosome 4, state 8, correspond to heterochromatin-like regions that occupy extensive domains in euchromatic arms of the chromosomes and contain lower levels of these chromatin marks. One of these regions, which was reported by Kharchenko et al as characteristic of this state, is shown in Figure 4B in this revised version of the manuscript and our data show that H3T3ph is enriched at this heterochromatin-like region. Moreover, we performed bioinformatics analyses using overlap permutation tests to statistically assess the location of H3T3ph in chromatin state 8. These analyses are now presented in Figure S7B and S7C. On the other hand, our ChIP-seq assays also show that H3T3ph enriched regions in chromosome arms tended to localize at state 9, which has been shown to be enriched in lamin by Kharchenko et al. [33]. H3T3ph localization in state 9 was also statistically assessed (Figure S7C).

This data and our H3T3ph immunofluorescence assays in polytene chromosomes are in our opinion the best evidences that this is not just the known mitotic centromeric H3T3ph signal.

8. P. 3. There is very good evidence that H3S10ph is not required for chromosome condensation, particularly in mammals. This sentence should be rephrased.

We are thankful to the referee for his/her advice and we have eliminated the sentence.

9. P.4. Haspin has not been shown to regulate cohesion in fission yeast.

We are thankful to the referee for his/her remark and we have rephrased the sentence.

In this revised version of the manuscript numerical data that underlies graphs is provided in spreadsheet form as supporting information.
